# Efficacy and safety of ciprofol for long-term sedation in patients receiving mechanical ventilation in ICUs: a prospective, single-center, double-blind, randomized controlled protocol

**DOI:** 10.3389/fphar.2023.1235709

**Published:** 2023-08-21

**Authors:** Xiaoxiao Sun, Meixian Zhang, Hui Zhang, Xuejie Fei, Gang Bai, Cheng Li

**Affiliations:** Department of Anesthesiology and Perioperative Medicine, Shanghai Key Laboratory of Anesthesiology and Brain Functional Modulation, Clinical Research Center for Anesthesiology and Perioperative Medicine, Translational Research Institute of Brain and Brain-Like Intelligence, Shanghai Fourth People’s Hospital, School of Medicine, Tongji University, Shanghai, China

**Keywords:** ciprofol, endotracheal intubation, long-term sedation, mechanical ventilation, propofol

## Abstract

**Introduction:** Critically ill patients who receive mechanical ventilation after endotracheal intubation commonly experience discomfort and pressure. The major sedative drugs that are currently used in clinical practice present with many complications, such as hypotension, bradycardia, and respiratory depression. Ciprofol (HSK3486), which is a newly developed structural analog of propofol, is a short-acting gamma-aminobutyric acid (GABA) receptor agonist, and its mechanism of action is sedation or anesthesia by enhancing GABA-mediated chloride influx. The high efficacy of ciprofol for short-term sedation is comparable to that of propofol, and it has a relatively low incidence of adverse effects and high level of safety, which has been confirmed by multiple clinical studies. However, few studies have examined its safety and efficacy for long-term sedation. The purpose of the study is to evaluate the efficacy and safety of ciprofol for long-term sedation in mechanically ventilated patients.

**Methods:** A prospective, single-center, double-blind, randomized, propofol-controlled, non-inferiority trial is proposed. The study will enroll 112 mechanically ventilated patients hospitalized in the intensive care unit (ICU) of the Shanghai Fourth People’s Hospital affiliated with Tongji University based on the inclusion and exclusion criteria of the study, and randomly assign them to a group sedated with either ciprofol or propofol. The primary outcome is the percentage of time spent under target sedation, and secondary outcomes include drug dose, number of cases requiring additional dextrometropine, incidence of systolic blood pressure <80 or >180 mmHg, incidence of diastolic blood pressure <50 or >100 mmHg, incidence of heart rate <50 beats per minute (bpm) or >120 bpm, inflammatory indicators, blood lipid levels, liver and kidney functions, nutritional indicators, ventilator-free days within the 7-day period after enrollment, 28-day mortality, ICU stay duration, and hospitalization costs.

**Discussion:** We hypothesize that the efficacy and safety of ciprofol for long-term sedation in mechanically ventilated ICU patients will not be inferior to that of propofol.

**Trial registration:** Chinese Clinical Trials Registry identifier ChiCTR2200066951.

## 1 Introduction

Critically ill patients on mechanical ventilation after endotracheal intubation are commonly admitted to intensive care units (ICUs) and typically experience stress states, such as pain, anxiety, and irritability. Such discomfort can stimulate the sympathetic nervous system and increase the risk of the patient removing their endotracheal tubes and intravascular catheters (Pun et al., 2019; Prabhakar et al., 2021; Temesgen et al., 2021). Guidelines recommend that mechanically ventilated patients should receive moderate analgesia and sedation to reduce anxiety and decrease their discomfort and psychological pressure (Devlin et al., 2018). Sedation and analgesia can reduce patient-machine disharmony events as well as decrease oxygen consumption and cardiovascular events, thereby reducing the incidence of secondary complications (Guerin, 2020). The study by Brook et al. (1999) showed that goal-directed sedation reduces the duration of mechanical ventilation and ICU stay as well as the need for tracheotomy in critically ill patients with acute respiratory failure. Therefore, moderate analgesic and sedative treatment must be used for patients in clinical practice.

The drugs that are most commonly used for sedation in clinical practice include benzodiazepines, propofol, and dexmedetomidine (Moller et al., 2022), and the most common complications include hemodynamic instability such as hypotension, bradycardia, and delirium; respiratory depression; bowel obstruction; renal impairment; venous return stasis; and immunosuppression (Zaal et al., 2015; Foster, 2016; Devlin et al., 2018; Duprey et al., 2021). Propofol is a gamma-aminobutyric acid (GABA) receptor agonist that acts as a sedative. It has a rapid onset of action and metabolism, and the most common adverse effects of propofol include loss of airway reflexes, hypoventilation, apnea, and hypotension. When the infusion of propofol is prolonged, “propofol infusion syndrome” may occur, which is a rare but serious adverse effect that includes severe metabolic acidosis, rhabdomyolysis, hyperkalemia, and cardiovascular failure that is usually fatal (Sahinovic et al., 2018). Ciprofol (HSK3486), a newly developed structural analog of propofol, is a short-acting GABA receptor agonist. Its mechanism of action is sedation or anesthesia by enhancing GABA-mediated chloride influx (White, 1989). It can be used in patients during invasive endoscopy and intensive care due to its sedative effects (Teng et al., 2021a). A clinical trial in Australia (Teng et al., 2021b) showed that ciprofol was safe at doses of 0.15–0.90 mg/kg, and most of the adverse effects of ciprofol were mild to moderate. In another clinical trial (Teng et al., 2021a), ciprofol was found to be safe at doses of 0.4–0.9 mg/kg, and it showed similar onset and duration of action as propofol, along with 4–5 times the potency of propofol. Studies have also found that in elderly patients undergoing painless gastroscopy, 0.2 mg/kg ciprofol could provide a sedative effect similar to that of 1 mg/kg propofol; moreover, it showed no significant differences in the induction and recovery times, and had fewer adverse reactions such as hypotension, respiratory depression, and injection pain as compared to the propofol group (Chen X. et al., 2022; Chen B. Z. et al., 2022; Li et al., 2022). Owing to its high efficacy, the dose used for clinical application as well as the incidence of adverse effects are lower than those for propofol. In conclusion, the efficacy of ciprofol for short-term sedation was comparable to that of propofol, and a relatively lower incidence of adverse effects was observed, as confirmed by an increasing number of clinical studies.

However, clinical studies on the efficacy and safety of the long-term use of sedatives in patients admitted to the ICU are limited. Based on previous findings, this study aims to investigate the efficacy and safety of ciprofol for long-term sedation over 7 days in patients undergoing mechanical ventilation and light sedation [Richmond Agitation-Sedation Scale (RASS) −3∼0].

## 2 Study design

This is a prospective, single-center, double-blind, randomized, propofol-controlled, non-inferiority study (Chinese Clinical Trials Registry identifier: ChiCTR2200066951) that will be conducted in accordance with clinical trial protocols (and any amendments), the Declaration of Helsinki (as currently revised), Chinese adult ICU analgesic and sedative treatment guidelines, and Clinical Practice Guidelines for the Management of Pain, Agitation and Delirium in Adult Patients in the ICU.

### 2.1 Study setting

**FIGURE 1:**
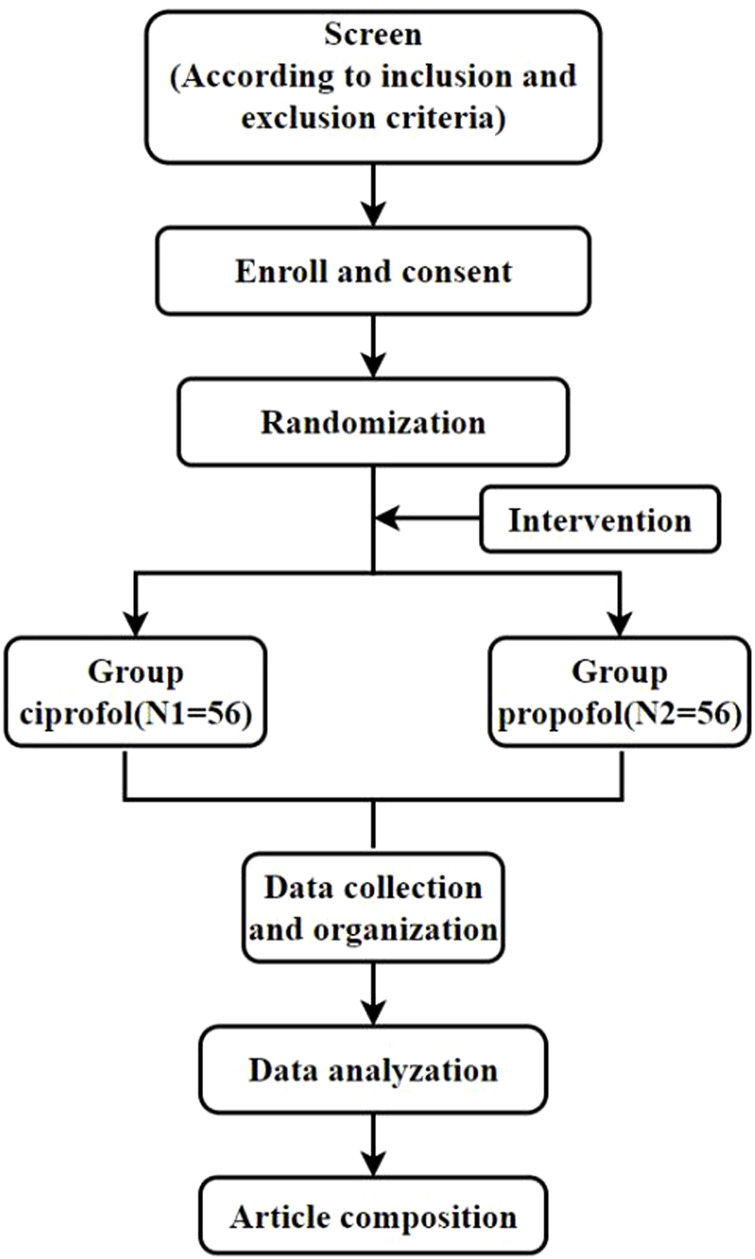
Technical route.

The study will enroll 112 patients undergoing mechanical ventilation and hospitalized in the ICU of Shanghai Fourth People’s Hospital affiliated with Tongji University, based on the inclusion and exclusion criteria of the study. These patients will then be randomly assigned to either ciprofol or propofol sedation groups. The technical scheme is shown in Figure 1.

### 2.2 Inclusion criteria

The inclusion criteria are as follows.

(1) Age, ≥18 years and ≤85 years; BMI, ≥18 kg/m^2^ and ≤30 kg/m^2^; either gender.
(2) Before enrollment, the patients would have been intubated and mechanically ventilated for no more than 96 h, and would have been scheduled to receive sedation for ≥24 h. The target sedation goal of patients will be within the range of RASS: −3∼0.
(3) The patients’ families must fully understand the purpose and significance of the trial, participate voluntarily, and sign an informed consent form.

### 2.3 Exclusion criteria

The exclusion criteria are as follows.

(1) Acute severe neuropsychiatric disease and various conditions that interfere with RASS.
(2) Systolic blood pressure at less than 90 mmHg with high doses of a single vasoactive drug to maintain blood pressure or two or more vasoactive drugs to maintain blood pressure.
(3) Heart rate less than 50 bpm, second- or third-degree atrioventricular block, and no pacemaker.
(4) Cardiac function class IV (NYHA Association) or severe cardiomyopathy.
(5) Severe liver dysfunction or acute liver failure (Child-Pugh class C).
(6) Chronic alcohol or drug use (benzodiazepines, opioid or heroin).
(7) Known allergies to eggs, soy products, or propofol and contraindications to propofol, opioids, and their relief medications.
(8) Contraindications to deep sedation (moribund state or myasthenia gravis) or previous sedation accident.
(9) Pregnancy or lactation in women.
(10) Unsuitability for inclusion in the study for various reasons based on the investigator’s judgement.

### 2.4 Randomization and blinding

The patients will be uniformly coded as 1, 2, , 112 based on their names, and a seed number would be set as 20221031. Random numbers would be generated using the Stata 17.0 software uniform() function, and the patients will be sorted by these numbers. Thereafter, the patients would be divided into the ciprofol or propofol group using the group() function, and a grouping data file will be generated.

Since patients in the ICU are often critically ill, keeping the researcher blinded may be difficult. Therefore, the patients, research evaluator, and dedicated nurses would be blinded for this study and not allowed to communicate drug-related information of the study with each other. The researcher would calculate the patients’ initial and supplemental doses prior to dosing based on the grouping, and the research evaluator would be primarily responsible for the timing of dosing initiation, dose adjustment, and medication discontinuation and provide the investigator with appropriate information in a timely manner. During this process, the researcher would not disclose any drug-related information.

### 2.5 Study drug and timelines

All patients will be given a loading dose of ciprofol (Haisco Pharmaceutical Group Co., Ltd., China) or propofol (AstraZeneca, United Kingdom). The total time of drug administration (including loading dose and maintenance dose) will be at least 24 h ± 30 min, and the longest time would be no more than 7 days.

### 2.6 Intervention

The patients will be randomized 1:1 to receive sedation with either ciprofol or propofol. At baseline, the delirium status would be assessed using the Confusion Assessment Method (CAM)-ICU scale. The lung-protective ventilation strategy would be adopted as follows (Hafiz and Stahl, 2019): 1) VT: 6∼8 ml/kg [ideal body weigh (IBW)]; 2) plateau pressure: <30 cmH_2_O; 3) stress pressure: <15 cmH_2_O; and 4) reasonable positive end-expiratory pressure (PEEP): 5∼10 cmH_2_O.

Prior to drug administration, analgesics would be continuously administered according to a standardized procedure. During the maintenance period, the analgesic dose would be adjusted according to the Critical-Care Pain Observation Tool score. The baseline sedation level of each patient must reach RASS ≥2 before the administration of the study drug.

Ciprofol group: Patients would receive a loading dose of 0.1 mg/kg ciprofol intravenously within 5 min and then a maintenance dose of 0.3 mg/kg/h ciprofol via continuous pumping. The maintenance dose range of ciprofol would be 0.06–0.8 mg/kg/h. An additional dose of 0.05 mg/kg ciprofol would be allowed. The injection would will be 30 s to 1 min, and the administration time would be ≥2 min.

Propofol group: Patients would be given a loading dose of 0.5 mg/kg propofol intravenously for 5 min, followed by a maintenance dose of 1.5 mg/kg/h propofol via continuous pumping. The maintenance dose range of propofol would be 0.3–4 mg/kg/h. During this process, an additional dose of 0.25 mg/kg propofol would be allowed. The injection time would be 30 s to 1 min, and the administration time would be ≥2 min.

If the maximum dose of the study drug would not be sufficient for sedation, dexmedetomidine would be infused at a rate of 0.2–1.0 μg/kg/h.

### 2.7 Data collection

The following demographic information will be collected: sex, age, BMI, prior alcohol use, and underlying disease. The following clinical data will be collected: percentage of time at target sedation (defined as the time during which additional dexmedetomidine is not required within the target sedation range), incidence of adverse events, ventilator-free days within 7 days after enrollment, 28-day mortality, ICU stay time, hospital costs, and laboratory indicators (inflammatory parameters, lipid parameters, liver function, renal function, and nutritional parameters before study drug administration and the day after study drug administration) (Table 1).

**TABLE 1:** Measurement schedule.

Time points	Baseline	Before study drug	Study drug intervention							Day after study drug ended	Discharged from hospital
			Day1 (per 4 h)	Day2 (per 4 h)	Day3 (per 4 h)	Day4 (per 4 h)	Day5 (per 4 h)	Day6 (per 4 h)	Day7 (per 4 h)		
Informed consent	×										
Inclusion/Exclusion criteria	×										
Demographic characteristics	×										
Medical History	×										
APACHE II	×										
SOFA	×										
Blood test		×								×	
Blood biochemistry		×								×	
Cytokines		×								×	
Blood coagulation		×								×	
Cardiac LVEF		×								×	
Remifentanil		×									
CPOT		×									
CAM-ICU		×									
RASS		×	×	×	×	×	×	×	×		
Dextrmetomidine (Yes/No)			×	×	×	×	×	×	×		
Adverse reactions			×	×	×	×	×	×	×		
ICU-stay time											×
Ventis											×
Hospital costs											×
28-day mortality											×

### 2.8 Trial termination

The following criteria will be used to indicate trial termination:

(1) Endotracheal tube is removed.
(2) Patient leaves the ICU.
(3) Physician discontinues treatment at 24 h.
(4) Period of 7 days after enrollment is concluded.

### 2.9 Sample size evaluation

This will be a randomized, controlled, non-inferiority trial. According to previous literature, the percentage of time in the target sedation state of propofol and ciprofol are 99.38% and 98.33% (Liu et al., 2022), respectively; however, the incidence of adverse events of ciprofol is lower than that of propofol. The sedative effect of ciprofol is not inferior to that of propofol. For a non-inferiority margin (*δ*) of 8%, set α to 0.025 (one side), test efficiency to 0.9, and the sample size of the two groups to be equal; then, the sample size of the test group and the control group is calculated by PASS 2021 software, with N1 = N2 = 50 cases. Considering that the loss to follow-up rate is 10%, 56 cases are required for both the test and control groups; thus, 112 patients will be enrolled in total to ensure the maintenance of the scientific design of the study.

### 2.10 Data management and statistical analysis

The person in charge of the study would explain how to complete the case report form. The data collector would complete the case report form according to the original medical records. A clinical supervisor will be responsible for verifying the integrity and authenticity of the data. The data administrator would be responsible for data entry.

SPSS software will be used for the statistical analyses. For continuous variables, Student’s *t*-test or the Mann–Whitney *U* test will be used based on the distribution. Examples of the mean, standard deviation, median, minimum, and maximum values will be listed. For categorical variables, the χ^2^ test will be used, and its frequency and percentage will be described.

### 2.11 Primary outcome

The primary outcome is the percentage of time in the target sedation state without other sedation drugs. When the lower limit of the 95% confidence interval of the mean difference between the two groups is lower than the negative limit value (−8%), then the study drug ciprofol would not be inferior to the control drug propofol.

### 2.12 Secondary outcome

Secondary outcomes will include the dosage of the study drug, number of cases with added dextrometropine, incidence of systolic blood pressure <80 or >180 mmHg, incidence of diastolic blood pressure <50 or >100 mmHg, incidence of heart rate <50 or >120 bpm, inflammatory indicators, blood lipid levels, liver and kidney functions, nutritional indicators, ventilator-free days within 7 days, 28-day mortality, ICU stay duration, and hospitalization costs.

## 3 Discussion

Propofol has advantages that include rapid onset and strong sedation. However, it can also cause adverse reactions, such as hypotension, respiratory depression, and propofol infusion syndrome, which is the most adverse reaction (Sahinovic et al., 2018). As a new sedative, ciprofol is expected to have the same sedation efficacy as propofol for ICU patients receiving long-term mechanical ventilation, with a lower incidence of adverse reactions caused by hypotension and drug accumulation than that of propofol. In a clinical trial, ciprofol was shown to be safe at a dose of 0.4–0.9 mg/kg, and it had similar onset and maintenance times as well as 4 to 5 times higher efficacy relative to propofol (Teng et al., 2021b). Due to its high efficacy, the dosage used in clinical applications can be reduced and the incidence of adverse reactions caused by drug accumulation would be lower than that of propofol. In a previous study on painless gastroscopy, the incidence of adverse effects such as hypotension and respiratory depression was lower in the propofol group than that in the control group. Further, early cognitive dysfunction was not observed after surgery (Chen X. et al., 2022). The study found that in painless gastroscopy of elderly patients, 0.2 mg/kg ciprofol can provide a sedative effect similar to that of 1 mg/kg propofol, and the induction time and recovery time did not differ significantly (Li et al., 2022). A phase 1 study of ICU patients (Teng et al., 2021b) showed that ciprofol as a 4- or 12-h infusion had good efficacy, rapid recovery, no significant accumulation, and an excellent safety profile.

In conclusion, this study will verify the efficacy and safety of ciprofol for long-term sedation in mechanically ventilated ICU patients and confirm whether it is inferior to propofol. We believe that ciprofol represents a new option for long-term sedation of ICU patients undergoing mechanical ventilation.

## Data Availability

The original contributions presented in the study are included in the article/Supplementary Material, further inquiries can be directed to the corresponding authors.
